# The Arabidopsis Protein Disulfide Isomerase Subfamily M Isoform, PDI9, Localizes to the Endoplasmic Reticulum and Influences Pollen Viability and Proper Formation of the Pollen Exine During Heat Stress

**DOI:** 10.3389/fpls.2020.610052

**Published:** 2020-12-29

**Authors:** Elizabeth Feldeverd, Brad W. Porter, Christen Y. L. Yuen, Kaela Iwai, Rina Carrillo, Tyler Smith, Cheyenne Barela, Katherine Wong, Pengfei Wang, Byung-Ho Kang, Kristie Matsumoto, David A. Christopher

**Affiliations:** ^1^Department of Molecular Biosciences and Bioengineering, University of Hawaii, Honolulu, HI, United States; ^2^State Key Laboratory of Agrobiotechnology, Centre for Cell and Developmental Biology, Chinese University of Hong Kong, Shatin, China

**Keywords:** protein folding, protein disulfide isomerase, heat stress, pollen viability, pollen exine biogenesis

## Abstract

Plants adapt to heat *via* thermotolerance pathways in which the activation of protein folding chaperones is essential. In eukaryotes, protein disulfide isomerases (PDIs) facilitate the folding of nascent and misfolded proteins in the secretory pathway by catalyzing the formation and isomerization of disulfide bonds and serving as molecular chaperones. In Arabidopsis, several members of the PDI family are upregulated in response to chemical inducers of the unfolded protein response (UPR), including both members of the non-classical PDI-M subfamily, PDI9 and PDI10. Unlike classical PDIs, which have two catalytic thioredoxin (TRX) domains separated by two non-catalytic TRX-fold domains, PDI-M isoforms are orthologs of mammalian P5/PDIA6 and possess two tandem catalytic domains. Here, PDI9 accumulation was found to be upregulated in pollen in response to heat stress. Histochemical staining of plants harboring the *PDI9* and *PDI10* promoters fused to the *gusA* gene indicated they were actively expressed in the anthers of flowers, specifically in the pollen and tapetum. Immunoelectron microscopy revealed that PDI9 localized to the endoplasmic reticulum in root and pollen cells. transfer DNA (T-DNA) insertional mutations in the *PDI9* gene disrupted pollen viability and development in plants exposed to heat stress. In particular, the pollen grains of *pdi9* mutants exhibited disruptions in the reticulated pattern of the exine and an increased adhesion of pollen grains. Pollen in the *pdi10* single mutant did not display similar heat-associated defects, but *pdi9 pdi10* double mutants (DMs) completely lost exine reticulation. Interestingly, overexpression of *PDI9* partially led to heat-associated defects in the exine. We conclude that PDI9 plays an important role in pollen thermotolerance and exine biogenesis. Its role fits the mechanistic theory of proteostasis in which an ideal balance of PDI isoforms is required in the endoplasmic reticulum (ER) for normal exine formation in plants subjected to heat stress.

## Introduction

Pollen has the most structurally complex cell wall produced by plants and serves as a highly protective barrier for the male gametophyte. The composition of the pollen cell wall changes throughout development and is precisely regulated by a transcription factor cascade (Xu et al., 2014; Lei et al., 2017), and the coordinated synthesis of biosynthetic enzymes secreted from the endoplasmic reticulum (ER; Lallemand et al., 2013). The pollen wall consist of two layers: an inner layer (intine) composed mainly of pectin and cellulose (Heslop-Harrison, 1968), and an outer layer (exine) that is primarily composed of the highly durable heterogeneous polymer, sporopollenin, which confers the pollen with formidable resistance to desiccation and degradation (Heslop-Harrison, 1968; Quilichini et al., 2015). Developing pollen is surrounded by a layer of highly metabolically active sporophytic anther cells designated the tapetum, which play a critical role in pollen wall biogenesis by synthesizing and secreting the sporopollenin monomers used to form the exine layer (Ahlers et al., 1999; Grienenberger et al., 2010; Quilichini et al., 2015; Battat et al., 2019). The tapetum also supplies the developing pollen cell with nutrients (Rieu et al., 2017), and regulatory molecules, such as phytohormones, which coordinate the process of pollen development (Sakata et al., 2010; Ye et al., 2010). The programmed cell death of the tapetum detaches it from the pollen cell wall, simultaneously depositing the final layer of sporopollenin (and in some species, lipid), causing the dehiscence of mature pollen grains from the anthers (Kawanabe et al., 2006; Parish and Li, 2010).

Pollen development is highly sensitive to abiotic stresses, such as heat, with exposure to elevated temperatures resulting in pollen cell death, male sterility, and a decrease in attachment to the stigma (Barnabás et al., 2008; Hedhly et al., 2009; Zinn et al., 2010; Katano et al., 2020). Pollen heat sensitivity is related to the increased demand for metabolic energy, and cell wall precursor and protein synthesis during the short phase of microspore formation and maturation into pollen grains (Lohani et al., 2019). Defects in the development of the tapetum serve as the primary basis for heat stress-induced male sterility in plants (De Storme and Geelen, 2014). During the course of pollen formation, the tapetum ER must maintain an increased capacity to synthesize, fold, and secrete proteins, sporopollenin biosynthetic enzymes and cofactors, and monomeric sporopollenin precursors (Fragkostefanakis et al., 2016). When proteostasis is jeopardized, such as upon exposure to heat stress (Rieu et al., 2017; McLoughlin et al., 2019), unfolded proteins accumulate to higher levels than can be dealt with by the folding capacity of the ER, leading to ER-stress (Howell, 2013). In turn, ER stress-sensing proteins and transcription factors activate the unfolded protein response (UPR; Nagashima et al., 2011; Lai et al., 2018). The UPR attempts to restore protein homeostasis by inducing protein folding catalysts and chaperones, while also temporarily downregulating some metabolic processes (Van Dalfsen et al., 2018). In addition, excess misfolded proteins are disaggregated to be folded (McLoughlin et al., 2019) or proteolytically degraded *via* ER-associated protein degradation (ERAD; Liu and Howell, 2016) through either the ubiquitin-proteosome pathway (ERAD-I; Book et al., 2010) or the autophagy/lysosome pathway (ERAD-II; Houck et al., 2014).

With the heavy demands placed on the ER, secretory apparatus, and protein folding processes, it is not surprising that the UPR is constitutively augmented in the tapetum in non-stressed conditions (Honys and Twell, 2003; Iwata et al., 2008). Moreover, studies on one of the three different ER membrane UPR sensors, inositol requiring enzyme-1 (IRE1), show that it affects pollen development in response to heat (Deng et al., 2016). The IRE1A/IRE1B complex is a ribonuclease-kinase (Chen and Brandizzi, 2012; Lai et al., 2018) that unconventionally splices *bZIP60* messenger RNA (mRNA) to produce the active form of the transcription factor, *bZIP60s* (Iwata et al., 2008; Nagashima et al., 2011), which subsequently activates the expression of downstream UPR genes. Interestingly, the IRE1A/IRE1B-mediated splicing of *bZIP60* mRNA is enhanced at elevated growth temperatures (Deng et al., 2011). Arabidopsis *ire1a ire1b* double knockout mutants (KOs) are fertile when grown at room temperature, but exhibit abnormal tapetum development, aberrant pollen coat deposition, and partial male sterility during heat stress, indicating that UPR plays an important role in pollen thermotolerance (Deng et al., 2016).

As part of the protein folding apparatus in the ER, protein disulfide isomerases (PDIs) catalyze the formation of disulfide bonds that stabilize the native conformations of many secretory proteins. The classical PDI, represented by the isoform PDIp in yeast and PDIA1 in mammals, exhibits both disulfide oxidoreductaste/isomerase and molecular chaperone activities (Noiva, 1999; Wilkinson and Gilbert, 2004), and has been shown to be essential for viability in both yeast (LaMantia and Lennarz, 1993) and nematodes (Winter and Page, 2000). In plants, the classical PDI5 functions as a protein foldase in the ER (Yuen et al., 2013), and a chaperone that inhibits cysteine proteases during trafficking to vacuoles, then releases the cysteine protease to engage in programmed cell death of the endothelium in developing seeds (Ondzighi et al., 2008). PDI2 mediates protein folding in the ER and localizes to both the secretory pathway and nucleus, where it interacts with maternal effect embryo arrest factor (Cho et al., 2011; Porter et al., 2015). In plants, yeast and animals, classical PDI isoforms are characterized by a domain arrangement consisting of two “*a*-type” catalytic thioredoxin (TRX) domains (*a*, *a'*), separated by two non-catalytic “*b*-type” TRX-fold domains (*b*, *b'*), in the sequence *a-b-b'-a'* (Kemmink et al., 1997). Due to their important role in the proper folding of proteins, the UPR activates the expression of several PDI genes in Arabidopsis (Lu and Christopher, 2008). Additionally, mammalian PDIA1 has been shown to be an activator of protein kinase R-like ER kinase (PERK), which mediates a UPR signaling pathway, the runs parallel to the IRE1/bZIP60 system (Kranz et al., 2017).

There are six structurally distinct PDI subfamilies in plants, designated PDI-A, -B, -C, -L, -M, and -S, with the members of the PDI-L subfamily sharing the classical PDI domain arrangement (Selles et al., 2011). The Arabidopsis genome encodes 14 members of the PDI family, of which six are transcriptionally upregulated by UPR *via* the IRE1/bZIP60 signaling pathway (Lu and Christopher, 2008). Among these UPR-induced genes are both members of the non-classical PDI-M subfamily, *PDI9* and *PDI10*. Moreover, the UPR-transcription factor *bZIP60* was shown to mediate *PDI9* transcription in response to UPR (Lu and Christopher, 2008). PDI-M isoforms possess two *a*-type domains and a single *b*-type domain, in the arrangement *a^o^-a-b*, with the tandem *a*-type domains often designated as *a^o^* and *a* to denote that they are not direct evolutionary counterparts to the *a* and *a'* domains of classical PDI. Molecular characterization of poplar PDI-M indicates that the *a^o^* domain preferentially catalyzes disulfide oxidation, while the *a* domain catalyzes reduction reactions (Selles et al., 2017). In rice grains, PDI-M strongly accumulates in ER-derived type I protein bodies (PB-I), and RNAi-mediated knockdown of the rice PDI-M gene *PDIL2;3* inhibits the accumulation of Cys-rich 10-kD prolamin in PB-I (Onda et al., 2011). When transiently expressed in Arabidopsis leaf protoplasts, green fluorescent protein (GFP) fusions of PDI9 and PDI10 also accumulate in ER-derived protein bodies, and the ER lumen (Yuen et al., 2013). Both PDI9 and PDI10 exhibit a strong capacity to catalyze disulfide bond formation, as demonstrated by their ability to functionally substitute for the major bacterial disulfide oxidase, DsbA, when heterologously expressed in an *Escherichia coli dsba^−^* mutant (Yuen et al., 2013).

Interestingly, a transcriptomic analysis of anthers revealed that the genes encoding PDI9 and PDI10, as well as the classical PDI-L isoform PDI5, are expressed at multiple stages of pollen development (Honys and Twell, 2003). Although these genes are upregulated as part of the UPR signaling pathway (Lu and Christopher, 2008), little is known concerning their role in pollen development or the UPR-mediated response to heat stress. Previously, the only Arabidopsis PDI associated with a pollen-related mutant phenotype was the PDI-S isoform, PDI11. Mutants expressing truncated versions of PDI11 exhibit disrupted pollen tube guidance, though *pdi11* null mutants do not display a similar phenotype, indicating that the truncation mutants are likely neomorphic (Wang et al., 2009).

In this study, we report that PDI9 is an ER protein that is highly expressed in anthers and pollen and is upregulated in response to heat. Insertional mutations in *PDI9* disrupt pollen viability and development in plants exposed to heat stress. In particular, the formation of the pollen exine is adversely affected, disrupting the reticulated pattern of the exine and leading to adhesion among pollen grains. Overexpression of *PDI9* also resulted in partially disrupted exine patterning of heat stressed pollen. We propose that PDI9 plays a crucial role in the ability of developing pollen to withstand heat stress, and that an ideal balance of specific PDI isoforms is required in the ER at elevated temperatures for proper exine formation and the overall maintenance of proteostasis within the secretory pathway.

## Materials and Methods

### Primers

The names and sequences of all primers used in this study are provided in Supplementary Figure 1.

### Plant Growth Conditions and Heat Stress Treatments

Except for the plants used in heat stress experiments, all Arabidopsis seeds were surface-sterilized and germinated on 0.5X Murashige and Skoog (MS) medium (Sigma-Aldrich) containing 1.5% (w/v) sucrose and solidified with 0.8% (w/v) Phytagel (Millipore-Sigma). For selectable marker screening, plants were sown horizontally on the agar medium containing the appropriate antibiotic, with a thin overlay of media also containing the antibiotic. All other seedlings were grown vertically on the agar surface. Liquid cultured plants were grown on the 0.5X MS, 1.5% sucrose medium without gelling agent. For plants grown in soil, Arabidopsis seedlings were initially grown on 0.5X MS, 1.5% sucrose medium, and then transferred at 1–2 weeks after germination to pots containing Fafard Super Fine Germinating Mix (Sun Gro Horticulture, Co. Agawam, MA, United States) supplemented with Miracle-Gro Plant Food (The Scotts Miracle-Gro Co.). Plants were grown at 22°C under a long-day photoperiod (16 h light/8 h dark cycle, 75 μmol photons m^−2^s^−1^). Various tissues were harvested from seedlings at 10–21 days except for flowers and siliques, for which plants were grown for 6–10 weeks, respectively.

For control and heat stress treatments, seedlings were grown on 0.5X Linsmaier and Skoog (LS) media (PhytoTechnology Laboratories Cat #: L473) supplemented with 3% sucrose for 7 days and transplanted to individual pots in a randomized design. All plants were initially grown at 22°C on a 16/8 h of light/dark cycle (approximately 75 μmol photons m^−2^s^−1^) for 2.5 weeks. Then, half of the plants were moved to a growth chamber for heat stress, while the other half (controls) remained in normal growth conditions. Heat stressed plants were exposed to 35°C 16 h light/27°C 8 h dark cycle according to (Pecinka et al., 2010), while the control plants remained at a constant 22°C. Pollen phenotyping took place after 2 weeks of heat stress. Siliques were returned to 22°C for 1–2 weeks to allow siliques to develop before phenotyping.

### Plant Materials

**Figure 1:**
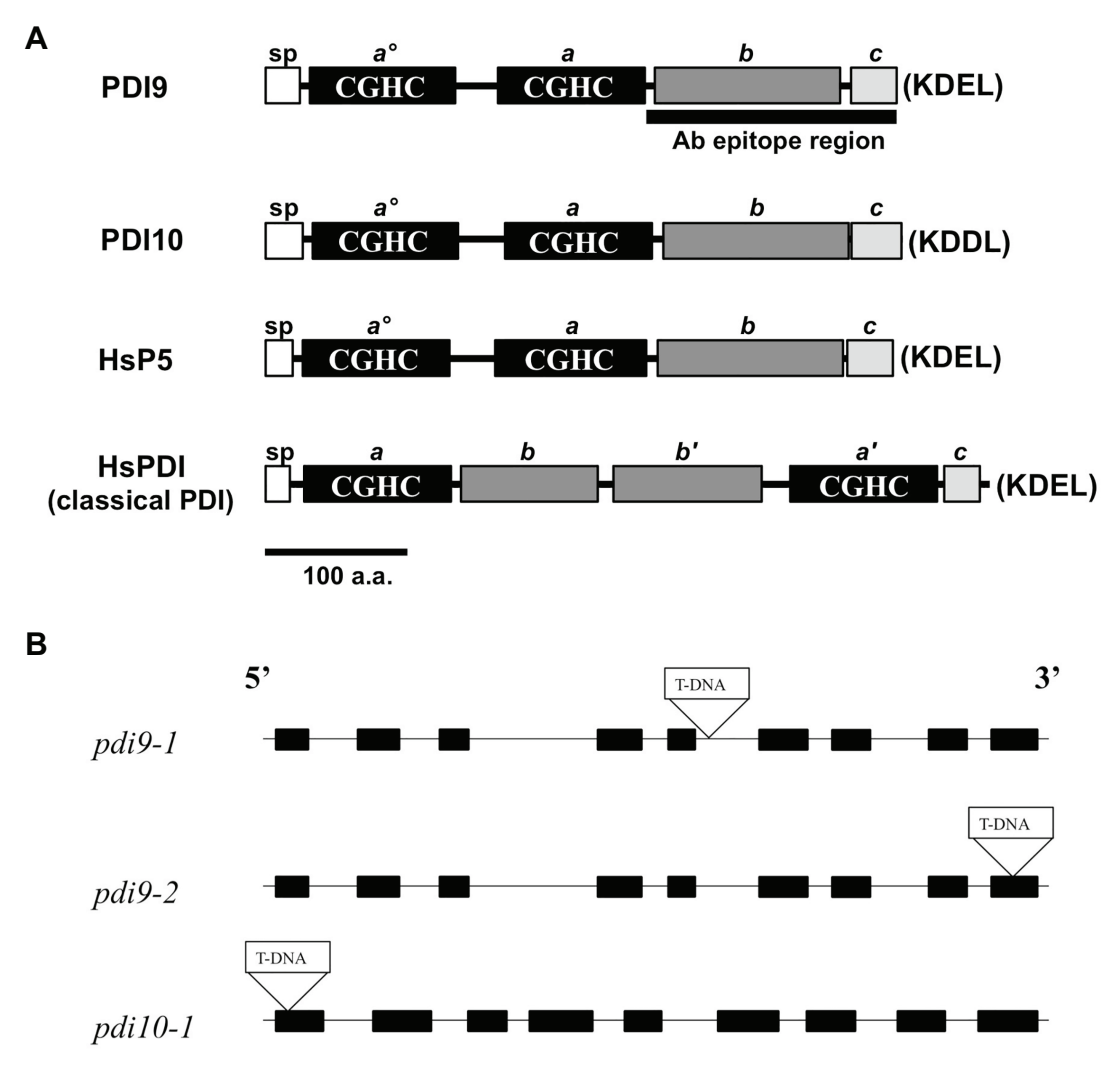
PDI-M subfamily protein domains and mutant gene maps. **(A)** The primary protein structures and domains of PDI-M subfamily members, PDI9 and PDI10, relative to the domain organization of mammalian classical PDI and P5. SP, signal peptide, thioredoxin (TRX) catalytic sites CGHC (termed *a^o^*, *a'*, and *a*), the TRX fold (*b*, *b'*), and acidic (*c*) domains. The last four residues of each protein (KDEL, KDDL), corresponding to potential ER retention signals, are shown in parenthesis. The black bar, “Ab epitope region,” denotes the less conserved subregion of the PDI9 protein used to make the specific antiserum. **(B)** The three independent transfer DNA (T-DNA) insertion sites are shown within the gene maps of the *pdi9* and *pdi10* loci, where black rectangles are exons and intervening connecting lines are introns.

*Arabidopsis thaliana* L. wild type Columbia (WT, Col-0) plants were used in this study, and all transfer DNA (T-DNA) insertion mutants and transgenic plants are in the Col-0 background. The *PDI9* and *PDI10* genes have the identifiers At2g32920 and At1g04980, respectively, as previously reported (Lu and Christopher, 2008). Three independent T-DNA insertion mutants were obtained from the Arabidopsis Biological Resource Center: *pdi9-1* (WiscDsLox445A08, progeny line CS864623 confirmed homozygous for the T-DNA), *pdi9-2* (GK-637C09), and *pdi10-1* (SALK_206219C). The position of the T-DNA insertion within each mutant allele is shown in Figure 1B. *PDI9* and *PDI10* both possess nine exons, and have gene lengths (from translation start to stop) of 2,905 and 2,252 bp, respectively. Relative to the translation start site, the positions of the T-DNA insertions are nt 1,612 (fifth intron) for *pdi9-1*, nt 2,821 (ninth exon) for *pdi9-2*, and nt 24 (first exon) for *pdi10-1*. The *pdi9 pdi10* double mutant (DM) was created by crossing *pdi9-1* × *pdi10-1* and identifying double homozygous mutant progeny in the F_3_ generation by PCR and sequencing. Genotyping at the *PDI9* locus was performed using primers PDI9g-F and PDI9g-R to detect the WT allele, *PDI9* primer PDI9-TDNA-Check-F (and double confirmed with primer PDI9g-F), and PDI-9-TDNA-Check-R (and double confirmed with primer WiscDsLoxP745), to detect *pdi9-1*, and *PDI9* primer PDI9g-F and T-DNA primer GK-8474 to detect *pdi9-2*. Genotyping of PDI10 was performed using primers PDI10g-F and PDI10g-R to detect WT *PDI10*, and PDI10-TDNA-Check-F2 (double confirmed with primer PDI10g_F) and LBb1 (double confirmed with primer Lba1) to detect *pdi10-1*. The absence of WT *PDI9* or *PDI10* mRNA in corresponding mutants was confirmed by reverse transcription PCR (RT-PCR), using primers PDI9g_F and PDI9g_R to detect the *PDI9* transcript, and primers PDI10rt_F1 and PDI10rt_1R to detect the *PDI10* transcript. Total mRNA for RT-PCR analyses were obtained from whole 7-day-old seedlings using the NucleoSpin RNA Plant kit (Machery-Nagel, Inc.).

The β-glucuronidase (GUS) reporter constructs for *PDI9* and *PDI10* promoter analysis were created by PCR amplification of the histological reporter gene, *gusA*, from pCAMBIA1304 using primers gusA-F-XhoI and gusA-R-BstEII, and the 5*'*-flanking sequences ~2.7 kb upstream of the start codons of *PDI9* and *PDI10* (containing the promoter regions) using the forward and reverse primers PDI9-Pr-F-KpnI and PDI9-Pr-R-NcoI for *PDI9*, and PDI10-Pr-F-KpnI and PDI10-Pr-R-NcoI-R for *PDI10*. The amplified sequences were inserted into the plant transformation vector pCAMBIA1302, with the *gusA* fragment digested with XhoI and BstEII and ligated between the vector restriction sites SalI and BstEII, and the *PDI9* or *PDI10* promoter fragment digested with KpnI and NcoI and ligated between the vector KpnI and NcoI restriction sites. The constructs were transformed into *Agrobacterium tumefaciens* strain GV3101, and then introduced into Arabidopsis (Col-0) plants by Agrobacterium-mediated transformation, using the floral dip method (Clough and Bent, 1998). The T_1_ transformants were screened for hygromycin resistance (Hyg^R^) by germinating seeds on ½ MS + 0.8% Phytagel media containing 50 μg/ml hygromycin B (Millipore-Sigma). The intactness of the promoter-GUS fusion transgene was assessed by PCR and sequencing of the PCR product. Segregation analysis of the Hyg^R^ marker was performed on T_3_ seedling populations to identify lines that were homozygous for the transgene (100% Hyg^R^). A total of 8–10 independent transgenic lines of each promoter-GUS fusion were selected for phenotype staining evaluation.

The *PDI9* overexpressor (OE) line, *35S:PDI9*, was created by replacing the GFP coding sequence of plant transformation vector pCAMBIA1302 with the *PDI9* cDNA sequence. The *PDI9* cDNA was amplified by PCR with primers, PDI9-F-PciI and PDI9-R-BstEII, purified and sequentially digested with the restriction enzymes PciI and BstEII, and ligated into the BstEII and NcoI sites of pCAMBIA1302, placing the *PDI9* coding sequence under the control of the Cauliflower Mosaic Virus (CaMV) 35S promoter. The T_1_ transformants were screened for Hyg^R^ by germinating seeds on ½ MS + 0.8% Phytagel media containing 50 μg/ml hygromycin B, and segregation analysis performed on the T_3_ generation to identify homozygous populations.

### Histochemical GUS Staining

β-glucuronidase staining was performed as described (Kim et al., 2006). Briefly, the tissue samples were fixed in 90% ice-cold acetone for 20 min at 25°C, then washed with staining buffer [50 mM sodium phosphate buffer (pH 7.0), 0.2% Triton X-100, 2 mM potassium ferrocyanide, and 2 mM potassium ferricyanide] three times on ice, then submerged in staining buffer containing 1 mM 5-bromo-4-chloro-3-indoxyl-*β*-D-glucuronide cyclohexylammonium salt (X-gluc). The tissues were vacuum infiltrated briefly, and then incubated O/N at 37°C. After staining, the samples were incubated in 70% EtOH to extract soluble pigments. Images of GUS staining in floral buds were taken on an Olympus SZX-12 stereomicroscope. For inflorescence staining, tissue samples were collected from 6-week-old *PDI9_promoter_:GUS* and *PDI10_promoter_:GUS* flowering plants.

### PDI9 Antiserum Production and Immunoblot Analysis of Plant Tissues

An affinity-purified polyclonal rabbit antiserum specifically recognizing PDI9 was generated commercially through YenZym Antibodies, LLC, using a truncated version of PDI9 (Figure 1) as the antigen for both rabbit immunization and affinity purification of the antiserum. For production of recombinant tcPDI9 protein, a cDNA fragment encoding a 173 amino acid residue portion of the C-terminus of PDI9 (residues Val268-Leu440) was amplified with the following primers: tcPDI9-Nde-for and tcPDI9-BH1-rev (Supplementary Figure 1). The resulting PCR fragment was digested with NdeI and BamHI, purified on a GFX column (Amersham Biosciences) and was ligated into the NdeI and BamHI sites of the bacterial expression vector pET15b (EMD Millipore). The tcPDI9 sequence was placed in-frame with the 6X His-tag of pET15b and verified by DNA sequencing. Expression of tcPDI9 was induced in *E. coli* strain BL21(DE3) for 3 h at 28°C by the addition of 1 mM isopropyl b-D-1-thiogalactopyranoside (IPTG). After induction, the *E. coli* cells were harvested by centrifugation and lysed using BugBuster Protein Extraction Reagent (EMD Millipore). The His-tagged tcPDI9 protein was purified from the lysate by nickel-nitrilotriacetic acid (Ni-NTA) affinity chromatography (EMD Millipore).

A 40 mg aliquot of total cell protein extracts from *E. coli* harboring either the pET-PDI9 expression construct or the pET-15b empty vector, with or without IPTG induction, were loaded on 10% polyacrylamide gels. The proteins were resolved by SDS-PAGE and transferred to Amersham Protran nitrocellulose membranes (GE Healthcare Life Sciences) by electroblotting. Immunoblot analyses were performed using the anti-PDI9 antibody at 1:500 dilution and a horseradish peroxidase (HRP)-conjugated anti-rabbit IgG secondary antibody at 1:3,000 dilution. The anti-rabbit secondary antibody and the reagents for chemiluminescent detection of HRP were supplied in the Amersham ECL Western Blotting Detection Kit (RPN2108; GE Healthcare Life Sciences).

For immunoblot analyses, proteins were isolated from the various plant tissues according to Martínez-García et al. (1999), from the WT, a constitutive *35S:PDI9* OE line, and the *pdi9–1* T-DNA mutant. For experiments demonstrating the absence of PDI9 protein in *pdi9* mutants, proteins were extracted from 7-day-old WT, *pdi9-1*, *pdi9-2*, *pdi9 pdi10* double mutant, and *35S:PDI9* seedlings. For tissue-specific expression studies, roots were obtained from 2-week-old seedlings grown in liquid culture under constant agitation. All other tissues were obtained from soil-grown plants, including stems and rosette leaves (4-week-old), flower and pollen (6-week-old), and siliques (8–12-week-old). Comparison of PDI9 abundance in normally grown and heat stressed plants was performed using protein extracted from the pollen of 6-week-old plants. The proteins (10 mg/lane) were electrophoresed on a 10% acrylamide SDS-PAGE gel, were electro-transferred to Amersham Protran nitrocellulose membranes (Amersham) and analyzed using the polyclonal rabbit PDI9-specific antiserum (1:500 dilution; except 1:100 for pollen samples) followed by an anti-rabbit HRP-conjugated secondary antibody at 1:2,000 dilution. The anti-rabbit secondary antibody and the reagents for chemiluminescent detection of HRP were supplied in the Amersham ECL Western Blotting Detection Kit (RPN2108; GE Healthcare Life Sciences). As a negative control, the PDI9-specific antiserum was substituted with pre-immune serum obtained from the host rabbit. Coomassie staining and probing with polyclonal rabbit anti-actin antibody (#SAB4301137, Millipore Sigma, Inc.) were performed as protein loading controls.

### Immunolabeling of Ultrathin Sections and Transmission Electron Microscopy Analysis

For roots, Arabidopsis seedlings were grown on 0.6% Phytoagar-solidified 0.5X MS medium at 22°C under continuous light. For anthers, Arabidopsis plants were grown for 6 weeks as described above under “Plant Growth Conditions.” High-pressure freezing, freeze substitution, and immunogold labeling were carried out according to Kang et al. (2011). In brief, root tips were excised from 5-day-old seedlings, whereas anthers and pollen were taken from floral buds of 42-day-old plants. Tissues were frozen rapidly with a HPM100 high-pressure freezer (Leica Microsystems, Buffalo Grove, IL, United States). Frozen tissue samples were freeze-substituted in anhydrous acetone containing 0.1% uranyl acetate and 0.25% glutaraldehyde at −80°C and embedded in Lowicryl HM-20 acrylic resin (Ted Pella, Inc., Redding, CA, United States). About 100 nm thick sections were prepared from wild-type and *35S:PDI9* tissue samples as described (Kang et al., 2011) and immunolabeled with the anti-PDI9 antibody. Then, they were labeled with 15 nm gold conjugated goat anti-Rabbit IgG (H + L; #15727; Ted Pella, Inc.). TEM images were collected with a Hitachi H-7650 Transmission Electron Microscope at 100 kV (Hitachi America, Inc., Schaumburg, IL, United States).

### High-Throughput Modified Alexander Viability Staining of Pollen

Alexander staining was performed as described by Peterson et al. (2010). Individual flowers at stage 13 (anthesis; Smyth et al., 1990) were harvested and placed in 1.5 ml microtubes, and 150 ml of Alexander staining solution (Peterson et al., 2010) was added immediately. The tubes were vortexed on high for 1 min to release the pollen from anthers, and then centrifuged for 2 min at 18,000 *g*. Flowers were carefully removed with a fresh pipette tip to avoid disturbing the pollen pellet. Hundred milliliter of solution was removed and 750 ml of ddH_2_0 was added followed by brief vortexing (10 s) and centrifugation for 1 min at 18,000 *g*. This wash step was repeated once more with fresh 750 ml ddH20. After the last wash, all but 25 μl of solution was removed. Pollen was resuspended in the 25 μl and was used for imaging.

The pollen sample was resuspended in the remaining 25 μl of solution and was added to glass microscope slides with 1.6 mm deep wells (Electron Microscopy Sciences Cat #: 71878-01) for imaging. The slides were examined under an Olympus BHB light microscope and photographed using with an AmScope WF200 camera. Pollen was counted from photographs at 4X or 10X using the image analysis program FIJI with a custom Javascript utilizing FIJI macros. Each image was converted to a 16-bit image using default thresholds, and pollen grains were identified from a defined minimum pixel size. Each pollen count was overlaid on the original RGB image. Aborted and clustered pollen grains were manually counted from the processed image. Each pollen grain is classified as non-aborted (viable), aborted (non-viable), or ambiguous. One-way ANOVA was performed for statistical analysis, calculated using Microsoft Excel.

### Scanning Electron Microscopy of Heat Stressed Pollen and Anthers

For qualitative imaging, anthers from stage 13 flowers from each genotype-treatment were harvested and gently dabbed directly onto the carbon mounting medium to release pollen. Pollen grains and anthers were gently moved on the mounting using a single hairbrush under a dissecting microscope and secured with conductive carbon tape (Electron Microscopy Sciences Cat. #: 77827-12) on aluminum stubs (Ted Pella Cat. #: 16111), then sputter-coated with gold/palladium (Anatech United States Cat. #: 1002021) in a Hummer 6.2 sputter apparatus. Specimens were viewed on a Hitachi S-4800 Field Emission Scanning Electron Microscope at an accelerating voltage of 5.0 kV at 4,000 and 11,000x magnifications.

## Results

### The PDI-M Subfamily Is Not Essential for Plant Viability Under Normal Growth Conditions

The two PDI-M genes of Arabidopsis, *PDI9* and *PDI10*, encode for highly similar proteins (79% sequence identity) with an identical domain arrangement consisting of two tandem catalytic domains (*a^o^*, *a*), followed by a non-catalytic TRX-fold domain (*b*), and a C-terminal acidic region (*c*; Figure 1A). This domain arrangement is shared by their mammalian ortholog, P5/PDIA6, and differs from the classical mammalian PDI domain arrangement, which consists of two catalytic domains (*a*, *a'*) separated by two central non-catalytic TRX-fold domains (*b*, *b'*), and a C-proximal acidic *c* region (Figure 1A). Note that, the term *b* domain is commonly used in the literature to indicate any domain possessing a TRX-fold but lacking redox activity, regardless of homology (Galligan and Petersen, 2012). Pairwise BlastP comparisons revealed that the *b* domains of PDI9 and PDI10 are homologous to the *b* domain of mammalian P5, but do not share significant sequence homology to the *b* or *b'* domains of classical mammalian PDI, or the *b*/*b'* domains of Arabidopsis PDI-B (Yuen et al., 2016) and PDI-L isoforms (Yuen et al., 2013). Whereas PDI9 and its mammalian ortholog P5 possess the C-terminal ER retention motif, KDEL, the C-terminus of PDI10 contains the variant sequence KDDL.

The *PDI9* and *PDI10* genes possess a similar arrangement of nine exons and eight introns, with exon and intron lengths being approximately similar between the two genes (Figure 1B). To gain further insight into the function of the PDI-M subfamily, T-DNA insertional mutants were obtained for both *PDI9* and *PDI10*. Using the SIGnAL T-DNA Express Arabidopsis Gene Mapping tool, two independent T-DNA alleles for *PDI9* were identified, located in intron 5 (*pdi9-1*) and exon 9 (*pdi9-2*), and a single T-DNA insertion mutant was identified for *PDI10* (*pdi10-1*), located 24 nt downstream of the translational start site in exon 1 (Figure 1B). The *PDI9* gene is the main subject of this study. Additionally, crosses were performed between *pdi9-1* and *pdi10-1* to obtain double mutants completely lacking functional PDI-M. And the two independent single *pdi9* mutants, *pdi9-1* and *pdi9-2*, were crossed to create the *pdi9-1*/*pdi9-2* transheterozygote hybrid mutant containing a single copy of each mutant gene, which was used as a test for allelism of the *pdi9-1* and *pdi9-2* mutant genes. The absence of wild-type *PDI9* or *PDI10* transcript in corresponding homozygous *pdi9-1*, *pdi9-2*, and *pdi10-1* plants and the transheterozygote mutant was confirmed by RT-PCR (Supplementary Figure 2).

No obvious phenotype was observed among the *pdi9* and *pdi10* single mutants, or *pdi9-1 pdi10-1* double mutant plants and the transheterozygote mutant, when grown under standard laboratory conditions (data not shown). Transgenic plants ectopically overexpressing *PDI9* under the CaMV 35S promoter (*35S:PDI9*) also did not display any obvious phenotypic differences when compared to wild-type plants grown in parallel (data not shown). Thus, PDI-M does not appear to be essential for plant viability, and loss of both isoforms in Arabidopsis does not result in an overt mutant phenotype under normal growth conditions.

### GUS Expression Analysis of the PDI-M Genes in Arabidopsis

**Figure 2:**
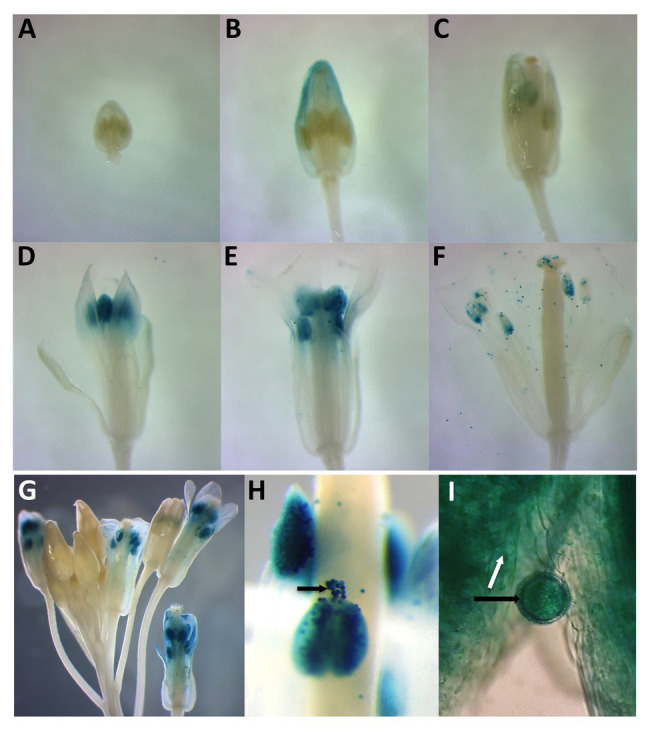
Histochemical analysis of two independent *PDI9_promoter_:*β-glucuronidase (*GUS*) and *PDI10_promoter_:GUS* expression constructs in developing and mature flowers. Panels **(A–F)** indicate a floral developmental series from 6-week-old plants expressing *PDI9_promoter_:GUS*. **(G)** Depiction of a large inflorescence expressing *PDI10_promoter_:GUS*. **(H)** Close-up view of *PDI10_promoter_:GUS* expression in anthers and pollen. **(I)** Close up of a pollen grain in *PDI9_promoter_:GUS* plants. Black arrows indicate pollen grains and white arrow the tapetum.

To compare the spatial expression patterns of the two PDI-M genes in various organs of Arabidopsis, multiple independent transgenic plants harboring the promoter sequences of *PDI9* and *PDI10* transcriptionally fused to the *gusA* reporter gene were generated. Histochemical staining of 14-day old *PDI9_promoter_:GUS* and *PDI10_promoter_:GUS* plants (Supplementary Figure 3) indicated that both promoters were active in petioles and leaves, with strong GUS staining observed in vascular tissue and apical meristem, and with weaker staining in sections of roots. GUS expression was also detected in trichomes (Supplementary Figure 3C). Most notably, in 6-week-old flowering plants, the *PDI9_promoter_:GUS* and *PDI10_promoter_:GUS* constructs are actively expressed during anther development in flowers (Figure 2), specifically during the formation of pollen grains and in the surrounding tapetum (Figures 2G–I). The prominent GUS staining in the pollen of post-anthesis flowers (floral development stage 13+) is consistent with transcriptomic data that indicated both *PDI9* and *PDI10* are expressed in pollen grains (Honys and Twell, 2003).

### Protein Expression Analysis and Subcellular Localization of PDI9

**Figure 3:**
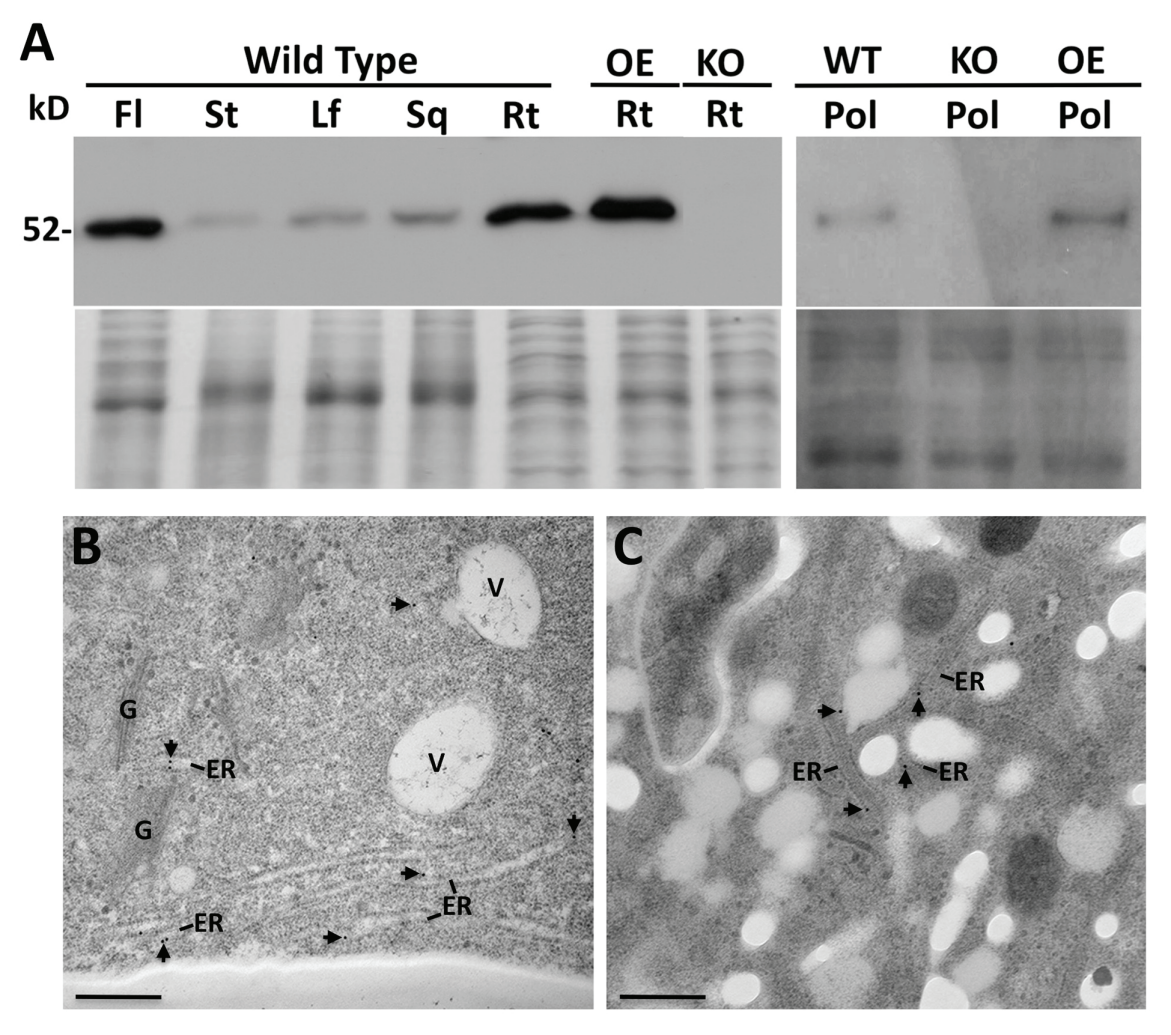
Immunoblot analysis using the PDI9-specific antiserum of proteins isolated from flowers (Fl), stems (St), Leaves (Lf), siliques (Sq), roots (Rt), and pollen (Pol), of Arabidopsis wild type (WT) and the *pdi9-1* single knockout mutant (KO) and the *35S:PDI9* overexpressor (OE) lines **(A)**. The 52 kD PDI9 protein band is denoted. The lower panel under the blot is the Coomassie stained protein gel. **(B,C)** Subcellular localization of PDI9 using TEM immunolabeling with the PDI9 antiserum (and 15 nm gold anti-rabbit secondary antiserum) on WT roots **(B)** and pollen **(C)**. Arrowheads point to immunogold particles associated with the ER. Other structures are indicated for reference, V, vacuole; G, Golgi apparatus. Scale bars are 500 nm.

A PDI9-specific antiserum was generated against the last 173 a.a. of PDI9, which encompasses the *b* domain and acidic *c* region, while omitting the redox-active *a^o^* and *a* domains (Figure 1A). The ability of the antibody to detect PDI9 was confirmed by immunoblot analysis of protein extracts obtained from 7-day-old WT, *pdi9-1*, *pdi9-2*, *pdi10-1*, and *pdi9-1 pdi10-1* double mutant seedlings, as well as the transgenic OE *35S:PDI9*. No protein band corresponding to PDI9 was detected for the mutant lines, *pdi9-1*, *pdi9-2*, or the *pdi9-1 pdi10-1* double mutants (27 and 60A), while the lane for *35S:PDI9* displayed noticeably more protein than WT (Supplementary Figure 2). Immunoblot analysis of proteins from various tissues of WT revealed that PDI9 was relatively abundant in flowers and roots, and was also present in rosette leaves, stems, and siliques (Figure 3A). The presence of PDI9 in pollen specifically was confirmed by immunoblot analysis of protein extracted from the pollen of WT plants, with *pdi9-1* and *35S:PDI9* serving as negative and positive controls, respectively (Figure 3A).

Due to the relatively high abundance of PDI9 in roots and flowers indicated by the immunoblots, the subcellular distribution of PDI9 was investigated in these tissues. Transmission electron microscopy (TEM) was performed on cryofixed thin tissue sections prepared from roots and anthers, which were immunolabeled with affinity-purified PDI9 antiserum and a 15 nm gold-conjugated anti-rabbit secondary antibody. The subcellular location of the PDI9 protein was determined to be in the ER in both roots (Figure 3B) and pollen (Figure 3C) of WT plants. No significant immunolabeling by the PDI9 antiserum was observed in the root cells of *pdi9-1* or the *pdi9-1 pdi10-1* double mutant, indicating that the antiserum exhibits minimal background cross-reactivity (Supplementary Figure 4). Although PDI-M isoforms were previously shown to highly accumulate in ER-derived protein bodies (Onda et al., 2011; Yuen et al., 2013), no labeling of such structures by the PDI9 antisera was observed in either roots or pollen. This likely reflects the relative absence of protein bodies in these tissues, which are most commonly associated with protein storage in the seed endosperm (Pedrazzini et al., 2016), or the sequestration of high levels of recombinantly expressed protein (Saberianfar and Menassa, 2017).

### Loss-of-Function Mutations of PDI9 Affect Pollen Viability During Heat Stress

**Figure 4:**
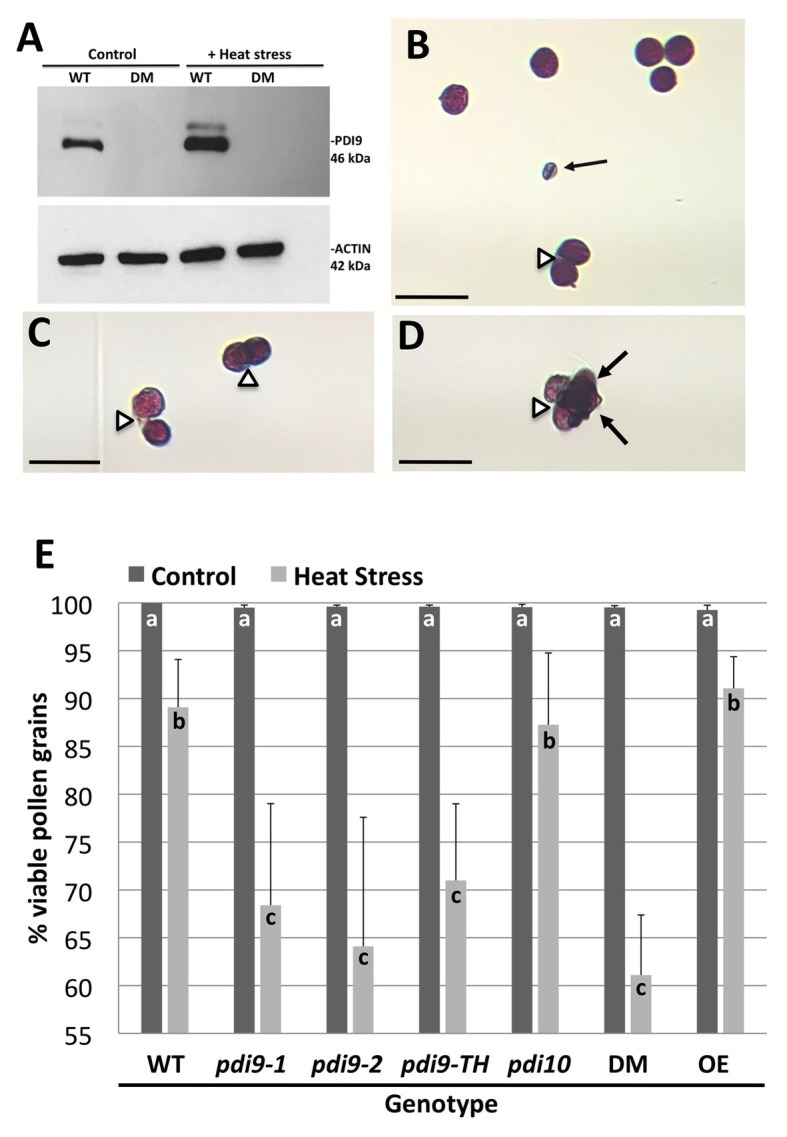
The expression of *PDI9* and the effects of *PDI9* and *PDI10* on the development of healthy pollen in plants exposed to heat stress. **(A)** Immunoblot analysis using the PDI9-antiserum on pollen proteins from WT, and the 27A *pdi9-pdi10* double mutant (DM), exposed to control and heat stress conditions. Alexander staining of pollen grains from WT **(B,C)** and DM **(D)** pollen from heat stressed plants. Black arrows point to aborted pollen grains, whereas open arrowheads indicate pollen grains sticking to each other. Scale bars denote 50 microns **(B–D)**. **(E)** Histogram showing the percent of total viable pollen grains for the single *pdi9* (two independent lines) and *pdi10* mutants, the *pdi9-pdi10* DM, *pdi9-1*/*pdi9-2* transheterozygote mutant line (*pdi9-TH*) and the PDI9 overexpressor line in untreated (control) and heat-stressed plants. Letters indicate significance between samples (*p* < 0.05 for *n* = 6 each genotype treatment for three independent experiments, except *pdi9-TH* which was two experiments).

The expression of PDI9 and PDI10 in anthers and pollen provided an opportunity to investigate the potential role of these protein folding catalysts in mediating plant responses to environmental stresses, such as heat stress, that perturb protein folding. Immunoblot analysis revealed that PDI9 abundance was substantially increased by 2.0-fold in heat-stressed pollen relative to pollen from plants grown under non-stressed conditions, with actin abundance serving as a protein loading control (Figure 4A). No PDI9 protein was detected in the *pdi9-1 pdi10-1* double mutant which served as a negative control (Figure 4A).

To determine if altered expression of PDI-M genes affects pollen sensitivity to heat stress, pollen viability under normal and heat stressed conditions was ascertained by Alexander’s viability staining. Under this assay, viable pollen grains are visually stained purple, whereas non-viable pollen, which is typically shriveled in appearance, are stained blue (Figure 4B). Notably, heat stress resulted in some pollen grains failing to properly separate from tetrads, leading to clusters of adhering viable and/or nonviable pollen grains (Figures 4B–D). Quantification of pollen viability under normal growth conditions revealed that the two independent homozygous *pdi9-1* and *pdi9-2* mutants, and *pdi10-1* mutants all possess pollen viability rates similar to WT (~99%), as do *pdi9-1*/*pdi9-2* transheterozygote mutant, *pdi9-1 pdi10-1* double mutants, and *35S:PDI9* transgenic OEs (Figure 4E). Upon heat stress, a significant decrease in viable pollen grains was observed across all genotypes. Statistically significant reductions in the average pollen viability percentages for *pdi9-1* (68%), *pdi9-2* (64%), *pdi9-1*/*pdi9-2* transheterozygote mutant (72%), and *pdi9-1 pdi10-1* double mutant plants (61%) were observed relative to pollen viability of WT plants (87%) grown under identical heat stress conditions (Figure 4E). On the other hand, the heat stressed pollen viabilities of the *pdi10-1* single mutant and *35S:PDI9* OE line did not differ significantly from WT.

The modified Alexander staining method described here combined with the ImageJ software allowed for the quantification of dehiscent pollen grains per flower (Table 1). Non-stressed Col-0 flowers at stage 13 (anthesis) have approximately 570–600 dehiscent pollen grains per flower, or 1,700–1,800 dehiscent pollen grains per sample (three flowers from one plant). Although all examined genotypes show a significant reduction in the number of dehisced pollen grains in plants exposed to heat stress (Table 1), the *pdi9* loss-of-function single mutants and *pdi9-1 pdi10-1* double mutant exhibited a significantly lower number of dehisced pollen grains (97–98% reduction) than WT (88% reduction) under heat stress. Surprisingly, the heat stressed *35S:PDI9* OE also displayed reduced pollen dehiscence relative to WT, though to a lesser extent than any of the *pdi9* mutants.

**Table 1:** Quantification of dehisced pollen grains for each genotype-treatment, including the two independent *pdi9* mutant lines.

Genotype	Control	Heat stress
WT	1918 ± 692^a^	277 ± 113^b^
*pdi9-1*	1,534 ± 454^a^	69 ± 40^d^
*pdi9-2*	1,572 ± 466^a^	69 ± 33^d^
*pdi10-1*	1,516 ± 450^a^	167 ± 74^bc^
*pdi9-pdi10* (*DM*)	1912 ± 700^a^	49 ± 21^d^
*pdi9-1*/*pdi9-2* (*TH*)	1,375 ± 433^a^	68 ± 49^d^
PDI9 OE	1,694 ± 689^a^	149 ± 71^c^

Unstressed control temperature and heat stressed pollen counts are from three stage 13 flowers per sample.

a-d indicate significance between samples (*p* < 0.05 for *n* = 6 per genotype-treatment).

### Effect of PDI9 Loss of Function Mutations on Pollen Morphology During Heat Stress

**Figure 5:**
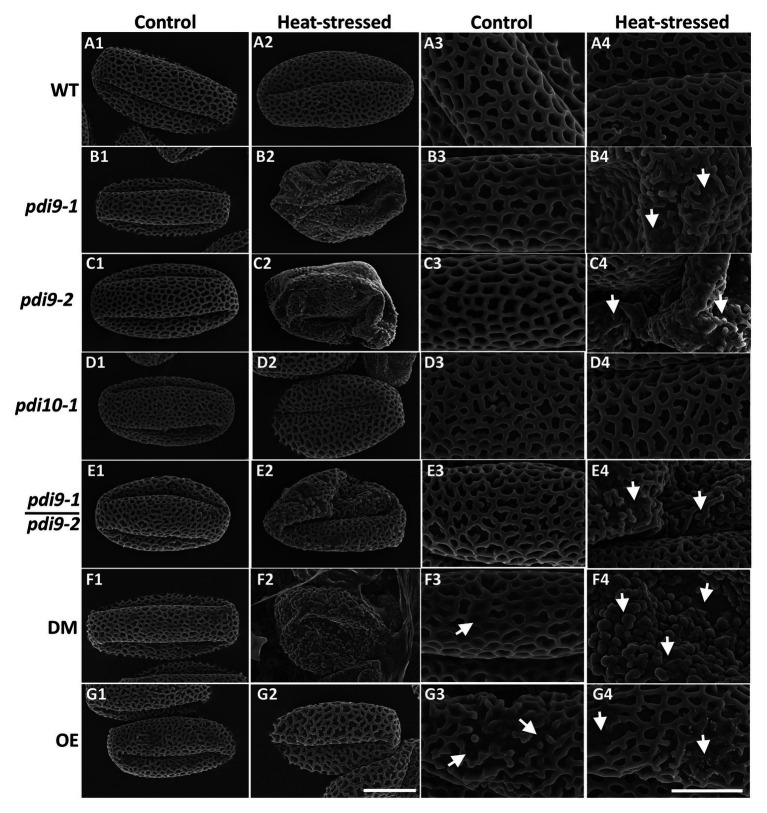
Analysis of the effects of PDI9 and heat stress on pollen and exine morphology by scanning electron microscopy (SEM). Each micrograph is a representative pollen grain from control (columns 1 and 3) and heat stressed (columns 2 and 4) plants. The *pdi9-1* and *pdi9-2* are two independent mutant lines (Figure 1A). WT row **(A)**, *pdi9-1* row **(B)**, *pdi9-2* row **(C)**, *pdi10-1* row **(D)**, the transheterozygote mutant (*pdi9-1*/*pdi9-2*) row **(E)**, the *pdi9-1 pdi10-1* DM row **(F)**, and the OE row **(G)** pollen grains were observed at either 4,000X (left half) or 11,000X (right half) magnification. The scale bar of 10 μm panel **(G2)** applies to all of the images in the left half, whereas the scale bar of 5 μm panel **(G4)** applies to the images in the right half.

To determine if mutations in PDI-M genes affect pollen morphology, scanning electron microscopy (SEM) was performed on pollen obtained from non-stressed (control) and heat stressed WT, *pdi9* single mutants (*pdi9-1*, *pdi9-2*) transheterozygote mutant (*pdi9-1*/*pdi9-2*), *pdi10-1* single mutant, *pdi9-1 pdi10-1* double mutant, and *35S:PDI9* OE plants. Pollen grains obtained from plants of each genotype grown in control conditions have essentially the same morphology consisting of three elongated colpi (furrows) and a reticulated surface pattern (column 1, Control Temperature, in Figure 5). The majority of pollen grains from heat stressed WT, *pdi10-1*, and *35S:PDI9* OE plants maintain a normal overall shape similar to that of non-stressed controls (Figures 5A2,D2,G2). In contrast, pollen grains from the *pdi9-1*, *pdi9-2*, *pdi9-1*/*pdi9-2* transheterozygote mutant, and *pdi9-1 pdi10-1* double mutants exposed to heat stress were severely deformed, having aberrant exine patterns (Figures 5B2,B4,C2,C4,E2,E4,F2) relative to pollen from non-stressed plants. The double mutant under heat conditions had the most disrupted exine pattern (Figures 5F2,F4) with no reticulation. Instead, various globular structures (possibly sporopollenin) formed on the exterior of *pdi9-1 pdi10-1* pollen grains (Figure 5F4). The *35S:PDI9* OE also has a moderately disrupted exine pattern, but unlike the double mutant, the defect is visible in control temperature as well as heat stressed pollen grains (Figures 5G3,G4). OE pollen grains have a combination of large patches of normal exine reticula with smaller areas of broken reticula (Figures 5G3,G4).

**Figure 6:**
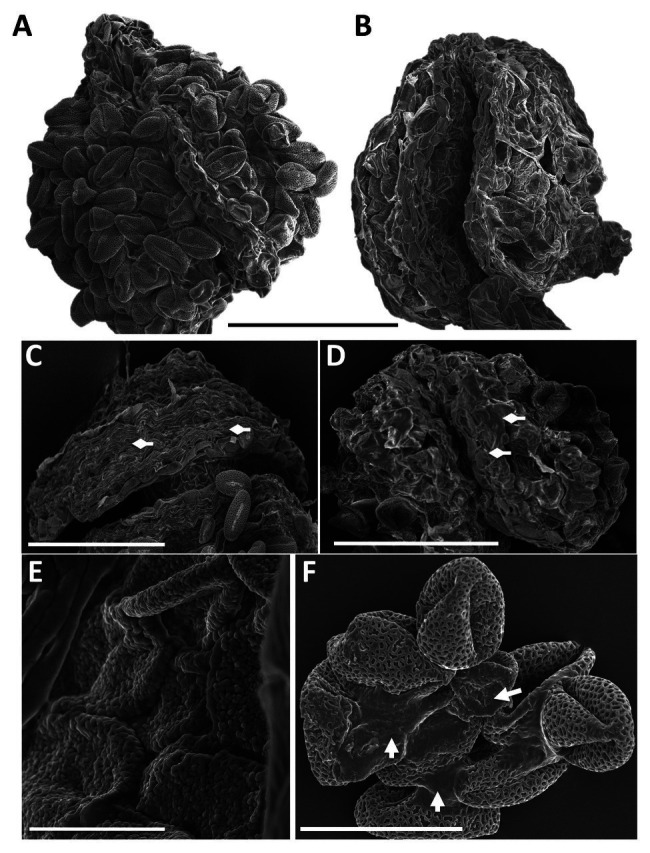
SEM analysis of anther morphology and pollen adhesion from flowers of WT **(A)**, the double mutant **(B,E)** and the transheterozygote *pdi9-1*/*pdi9-2* hybrid mutant **(C,D,F)** in the control **(C)** and heat stress treatments **(A,B,D-F)**. Scale bars are 100 mm **(A–D)**, 10 mm **(E)**, and 30 mm **(F)**. Diamond arrows indicate tapetum cells **(C,D)** and regular arrows indicate points of pollen adhesion **(F)**.

High magnification SEM images of whole anthers from WT (Figure 6A) and *pdi9-1 pdi10-1* double mutant (Figures 6B,E) plants exposed to heat stress revealed that the WT pollen had a distinct spherical shape (Figure 6A), whereas double mutant pollen were shriveled and stuck together in the anther (Figures 6B,E). Pollen adhesion or clustering was also seen in *pdi9* single mutants exposed to heat stress, with a representative image of a tranheterozygous *pdi9-1/pdi9-2* anther shown in Figure 6F. The areas of pollen adhesion are smooth, lacking a reticulate pattern. In addition, when the pollen was carefully removed before fixation to expose the underlying tapetum, differences were seen in the organization of tapetal cells in control (Figure 6C) relative to heat stressed flowers (Figure 6D). Tapetal cells from control anthers were organized in parallel striations, whereas in the heat stressed anthers, the cells were disorganized and fused. Based on mutant phenotype obtained with the transheterozygote mutant (Figures 5, 6), the two mutant genes *pdi9-1* and *pdi9-2* are allelic and fail to complement each other in the F1 hybrids. This further supports that the mutant heat stress phenotypes were caused by the disruption of the *PDI9* gene by the T-DNA, and not caused by a T-DNA located somewhere else in the genome. Taken together, the external analysis of the pollen grains and anthers indicated that the absence of PDI9 in the single mutants, and both PDI9 and PDI10 in the double mutant, dramatically disrupted the development of the external exine and release of the pollen during heat stress.

## Discussion

The plant PDI-M subfamily is orthologous to mammalian P5/PDIA6 and has previously been shown to accumulate in ER-derived type-I protein bodies (PB-I) and the ER lumen (Onda et al., 2011; Yuen et al., 2013). In rice, RNAi-mediated suppression of the PDI-M gene *PDIL2;3* impairs the accumulation of Cys-rich 10-kD prolamin in PB-I (Onda et al., 2011). In Arabidopsis, the PDI-M subfamily is part of the UPR pathway (Lu and Christopher, 2008). In this report, we have demonstrated a new function for this subfamily in facilitating the formation of viable pollen during heat stress.

Phenotypic characterization of mutants harboring T-DNA insertions in the two PDI-M genes of Arabidopsis, *PDI9* and *PDI10*, revealed that the pollen of *pdi9* loss-of-function mutants were hypersensitive to heat stress, as indicated by a pronounced decrease in pollen viability in comparison to WT control plants grown under identical conditions. Although GUS reporter experiments indicated that the promoters of both *PDI9* and *PDI10* were active in anthers and pollen, the pollen of *pdi10-1* single mutants did not exhibit a similar susceptibility to heat stress as the *pdi9-1* and *pdi9-2* mutants. Thus, PDI9 plays an important role in pollen tolerance to elevated temperatures, and its loss cannot be compensated for by the paralogous PDI-M isoform, PDI10. Interestingly, whereas PDI9 possesses the canonical C-terminal ER retention motif, KDEL, the protein sequence of PDI10 ends with the variant sequence KDDL, which has previously been shown to be ineffective as an ER retention signal in tobacco (Denecke et al., 1992). We speculate that the inability of PDI10 to functionally compensate for PDI9 may be due, at least in part, to differences in their abilities to be retained in the ER lumen.

At the morphological level, the pollen of *pdi9* mutants display several heat stress-associated phenotypes, including collapsed pollen grains, disruption of the sculpted reticulate pattern of the exine, and severe cohesion of pollen grains obviating dehiscence (Figures 5, 6). Interestingly, although *pdi10-1* single mutants do not display overt pollen defects under the heat stress conditions used in this study, *pdi9-1 pdi10-1* double mutants exhibit a more severe pollen wall phenotype than *pdi9* single mutants, including the absence of reticulation and appearance of globular structures on the pollen surface. Similar globular structures were observed in the pollen of *transient defective exine 1* (*tde1*) mutants, which also exhibit defects in exine formation, and were caused by the accumulation of sporopollenin aggregates (Ariizumi et al., 2008). The more severe pollen wall phenotype of *pdi9-1 pdi10-1* double mutants suggests that while PDI9 is required for normal exine patterning during heat stress, PDI10 has some capacity to fulfill this function, as indicated by the milder disruption to the exine patterning observed in *pdi9-1* and *pdi9-2* single mutant pollen.

The tapetum plays a key role in pollen development by nourishing the microspore and supplying the precursors utilized in pollen wall formation (Parish and Li, 2010; Ariizumi and Toriyama, 2011; Lallemand et al., 2013; Quilichini et al., 2015; Fragkostefanakis et al., 2016). Impairment of the tapetum secretory pathway can have a severe impact on proper wall formation and the viability of pollen (Lallemand et al., 2013; Quilichini et al., 2014). For example, KOs of the tapetally-expressed *SECRETORY31B* (*SEC31B*) gene, which encodes a COPII protein and is required for proper ER-Golgi protein trafficking, exhibit severely reduced pollen viability and aberrant pollen exine formation (Zhao et al., 2016). Similar pollen defects were observed in loss-of-function mutants of another tapetally-expressed gene, *MALE GAMETOGENESIS IMPAIRED ANTHERS* (*MIA*), which encodes a P-type ATPase cation channel pump that appears to be required for the proper secretion of vesicle cargo to the plasma membrane (Jakobsen et al., 2005). Recently, mutation in a secreted non-specific lipid transfer protein of the plasma membrane of meiocytes disrupted the unique exine pattern of rice pollen (Li et al., 2020).

Heat stress can lead to the accumulation of unfolded proteins, thereby triggering UPR as a means of restoring proteostasis within the secretory pathway. IRE1, a dual protein kinase/ribonuclease of the ER, is a key component of UPR, and the loss of both Arabidopsis IRE1 isoforms in *ire1a ire1b* double mutants causes male sterility and irregular exine patterning in plants grown under modestly elevated temperatures (Deng et al., 2016). Mutations in another UPR gene, *bZIP60*, which transcribes the direct mRNA splicing target of IRE1, are also associated with heat stress-induced pollen defects (Deng et al., 2016).

Given the role of PDIs as protein folding catalysts, we hypothesize that the heat-related pollen defects observed in *pdi9* mutants is the result of an impaired ability to respond to protein misfolding through the UPR pathway. This interpretation is further supported by the observation that PDI9 accumulation occurs in pollen in response to heat stress, as well as the strong connection of *PDI9* expression to the UPR pathway that was previously established in which *PDI9* transcription is upregulated in response to the UPR and this upregulation is mediated by the UPR-transcription factor, *bZIP60* (Lu and Christopher, 2008). However, aberrations in tapetum development and the timing of tapetum degeneration are also known to cause defects in pollen *via*bility and morphology (Rieu et al., 2017), and thus it is possible that the defect in tapetum cell organization of *pdi9* mutants during heat stress (Figure 6D) also contributes to the production of defective pollen.

While our characterization of Arabidopsis PDI-M mutants reveals an involvement in pollen tolerance to heat stress, it is interesting to note that GUS reporter experiments indicate that the promoters of both *PDI9* and *PDI10* are active in pollen grains under normal (non-stressed) growth conditions beginning at flower development stage 13 (anthesis). Although the UPR pathway is activated in response to stresses that cause protein misfolding, components of the UPR pathway are also known to be active during plant growth and development in the absence of external stresses, particularly in instances where there are heavy demands for secretion (Honys and Twell, 2003; Iwata et al., 2008). The expression of *PDI9* and *PDI10* in pollen during and after anthesis may indicate a role for PDI-M isoforms in the folding of proteins involved in dehiscence and the later stages of pollen maturation, or may reflect an increased load on the secretory pathway of pollen during these processes leading to the general activation of UPR.

Here we have shown that PDI9 contributes significantly to the thermal tolerance of Arabidopsis pollen, and loss-of-function mutants of *PDI9* display heat stress-related pollen phenotypes similar to those reported previously for UPR mutants (Deng et al., 2016). PDI9 is a member of the plant PDI-M subfamily, which has previously been implicated in the proper accumulation of storage proteins in rice (Onda et al., 2011). In animals, the zebrafish PDI-M ortholog PDI-P5 was revealed to be essential for specifying left/right asymmetries (Hoshijima et al., 2002). The disparate phenotypes among eukaryotes currently associated with the loss of PDI-M/P5 may reflect the numerous potential substrates for PDI-M within different organisms at various stages of development, both as a disulfide oxidoreductase/isomerase and a molecular chaperone (Kikuchi et al., 2002). Notably, the two identified PDI-M roles in plants are processes during which a heavy load is placed on the secretory pathway, and it may be the case that PDI-M genes are commonly expressed in plant cells undergoing rapid synthesis of proteins sorted through the secretory pathway. *In vitro*, mammalian P5 catalyzes rapid, but promiscuous disulfide bond formation in substrates, whereas classical PDI was found to be an efficient proofreader of incorrect disulfides (Sato et al., 2013). We hypothesize that a similar situation may occur in plants. PDI-M isoforms are expressed in plant cells producing large amounts of secretory proteins, or where misfolded proteins are present due to heat stress, to rapidly introduce disulfide bonds into these proteins. Subsequently, these disulfide bonds can be corrected into native disulfides, if necessary, by the proofreading action of classical-type PDI-L isoforms. The pollen defects observed in heat stressed *pdi9* mutants demonstrate that PDI-M fulfills a distinct role in plant protein folding that cannot be fully compensated for by other members of the PDI family.

Interestingly, aberrant exine formation was observed in heat stressed plants overexpressing PDI9 under the CaMV 35S promoter. We speculate that this could be the result of an increase in PDI9-mediated promiscuous disulfide bond formation, which outpaces the ability of proofreading “classical PDIs” to shuffle substrates into native disulfide arrangements *via* their isomerase activity. Therefore, the correct balance of PDI-M and PDI-L activities is necessary for proteostasis. Furthermore, there is precedent for a UPR protein to have a homeostatic or “Goldilocks” level of expression in pollen. The ER protein, Tunicamycin Induced 1 (TIN1), is induced during ER stress and is abundant in pollen (Iwata et al., 2010). Both knockout *tin1* and pollen grains from the 35S:TIN1 OE line have an altered pollen surface morphology, including an especially sticky pollen coat (Iwata et al., 2012, 2017). No enzymatic or structural function has been assigned to TIN1. It was suggested that TIN1 mutants have a defective ER quality control system in developing pollen, such that the misregulated secretion of proteins and perhaps lipids result in an abnormal exine (Iwata et al., 2017). They suggested there is an amount of TIN1 in pollen that is homeostatically just right for proper development, with either too much or a deficiency of TIN1 adversely affecting the exine. By analogy, *PDI9* overexpression and the *pdi9* mutants all have reduced pollen counts and, to various degrees, disrupted exine patterns relative to WT in the heat treatments. Therefore, the role of PDI9 in pollen development arguably fits the mechanistic theory of proteostasis (Taylor et al., 2014) in which an ideal balance of PDI-M and PDI-L activities is required in the ER for normal exine formation in the heat.

## Data Availability Statement

The original contributions presented in the study are included in the article/supplementary material, further inquiries can be directed to the corresponding author/s.

## Author Contributions

EF assisted in designing the study, performed experiments on heat stress, measured pollen viability, dehiscence, and protein levels, conducted scanning electron microscopy (SEM), analyzed data, and contributed to writing the manuscript. BP performed mutant crossing, selfing, and confirmation experiments, created the double mutant, conducted histochemical GUS staining, light microscopy, and contributed to editing the manuscript. CY created the GUS and 35S *PDI9* overexpressor lines and contributed to writing and editing the manuscript. KI performed experiments on heat stress, measured pollen viability, and conducted SEM. RC performed western blot analysis of plant tissues, characterized mutants, and wrote methodology. TS performed histochemical GUS staining and light microscopy. CB performed histochemical GUS staining, western blotting of plant tissues, and PCR. KW isolated the *PDI9* and *PDI10* promoters, and created the *PDI9*-Promoter‐ and *PDI10*-promoter GUS lines. PW and B-HK conducted immunolabeling transmission electron microscopy (TEM). KM subcloned the *PDI9* cDNA for recombinant *PDI9* expression and purification, generated the *PDI9* antiserum, and wrote methodology. DC conceived the experiments, planned and designed the study, supervised and coordinated the work, provided the grant funding, and contributed to writing the paper. All authors contributed to the article and approved the submitted version.

### Conflict of Interest

The authors declare that the research was conducted in the absence of any commercial or financial relationships that could be construed as a potential conflict of interest.
