# Supplementary material for: The Arabidopsis Protein Disulfide Isomerase Subfamily M Isoform, PDI9, Localizes to the Endoplasmic Reticulum and Influences Pollen Viability and Proper Formation of the Pollen Exine During Heat Stress
Source: Front Plant Sci. 2020 Dec 29;11:610052. doi: 10.3389/fpls.2020.610052 (PMC7802077; doi:10.3389/fpls.2020.610052)
Supplement: Supplementary file 1 [file Data_Sheet_1.pdf]

Supplementary Figure 1: Table of primers used for cDNA synthesis, cloning, genotyping and RT-PCR. Introduced restriction sites are shown in underlined italics.

**Primer**	**Sequence, 5' to 3'**
PDI9-F-PciI	TTTTT*<u>ACATGT</u>*ATAAATCACCATTAACGTTGCT
PDI9-R-BstEII	TTTTT*<u>GGTCACC</u>*TCACAACTCATCCTTAGAACCAACAG
gusA-F-XhoI	GGA*<u>CTCGAG</u>*ACCATGGTAGATCTGACTAG
gusA-R-BstEII	CTCC*<u>GGTCACC</u>*TATTGTTTGCCTCCCTGCTGCG
PDI9-Pr-F-KpnI	GTT*<u>GGTACC</u>*GTAGTTTGGATAGTTGATGCAC
PDI9-Pr-R-NcoI	TAT*<u>CCATGG</u>*TTCTTCCCCTTTCTCTC
PDI10-Pr-F-KpnI	TAT*<u>GGTACC</u>*AAGGTGATCGAAGAATTTC
PDI10-Pr-R-NcoI-R	TTT*<u>CCATGG</u>*TTTCTTCCCTCTCTTTCTTCT
tcPDI9-Nde-for	TCT*<u>CATATG</u>*GTGGAATCCAGTGCTGGCCCTG
tcPDI9-BH1-rev	TTG*<u>GGATCC</u>*TCACAACTCATCCTTAGAACCAAC
WiscDsLox445A08	AACGTCCGCAATGTGTTATTAAGTTG
WiscDsLoxP745	AACGTCCGCAATGTGTTATTAAGTTGTC
Lba1	TGGTTCACGTAGTGGGCCATCG
LBb1	GCGTGGACCGCTTGCTGCAACT
GK-8474	ATAATAACGCTGCGGACATCTACATTTT
PDI9g-F	ATGTATAAATCACCATTAACGTTGC
PDI9g-R	TCACAACTCATCCTTAGAACCAAC
PDI9 Frag3_F	CAAACTTGTGTACCTGCATTCAG
GK637CO9_R	CATATTGACCATCATACTCATTGCTGA
PDI10g-F	GCTTTGAAGGCACCAGAATGTGG
PDI10g-R	TATGGACAAACCTGCTTGATAGC
PDI10rt-F1	CAACTCACTCCTTCTAATTTCAAGTCTAAGGTTC
PDI10-TDNA-Check-F2	GGTTGGCGAGGCGGGATATGGTATGC
PDI10rt-R1	CCAGCATTGGATTCTAGCTGTTCCAG
PDI9-TDNA-Check-F	TGGATCTTCGTCACCTGTGGTTCAGC
PDI-9-TDNA-Check-R	ACCATTGCTGGATAACCGTATCCTCC
