# Supplementary material for: The Arabidopsis Protein Disulfide Isomerase Subfamily M Isoform, PDI9, Localizes to the Endoplasmic Reticulum and Influences Pollen Viability and Proper Formation of the Pollen Exine During Heat Stress
Source: Front Plant Sci. 2020 Dec 29;11:610052. doi: 10.3389/fpls.2020.610052 (PMC7802077; doi:10.3389/fpls.2020.610052)
Supplement: Supplementary file 2 [file Data_Sheet_2.pdf]

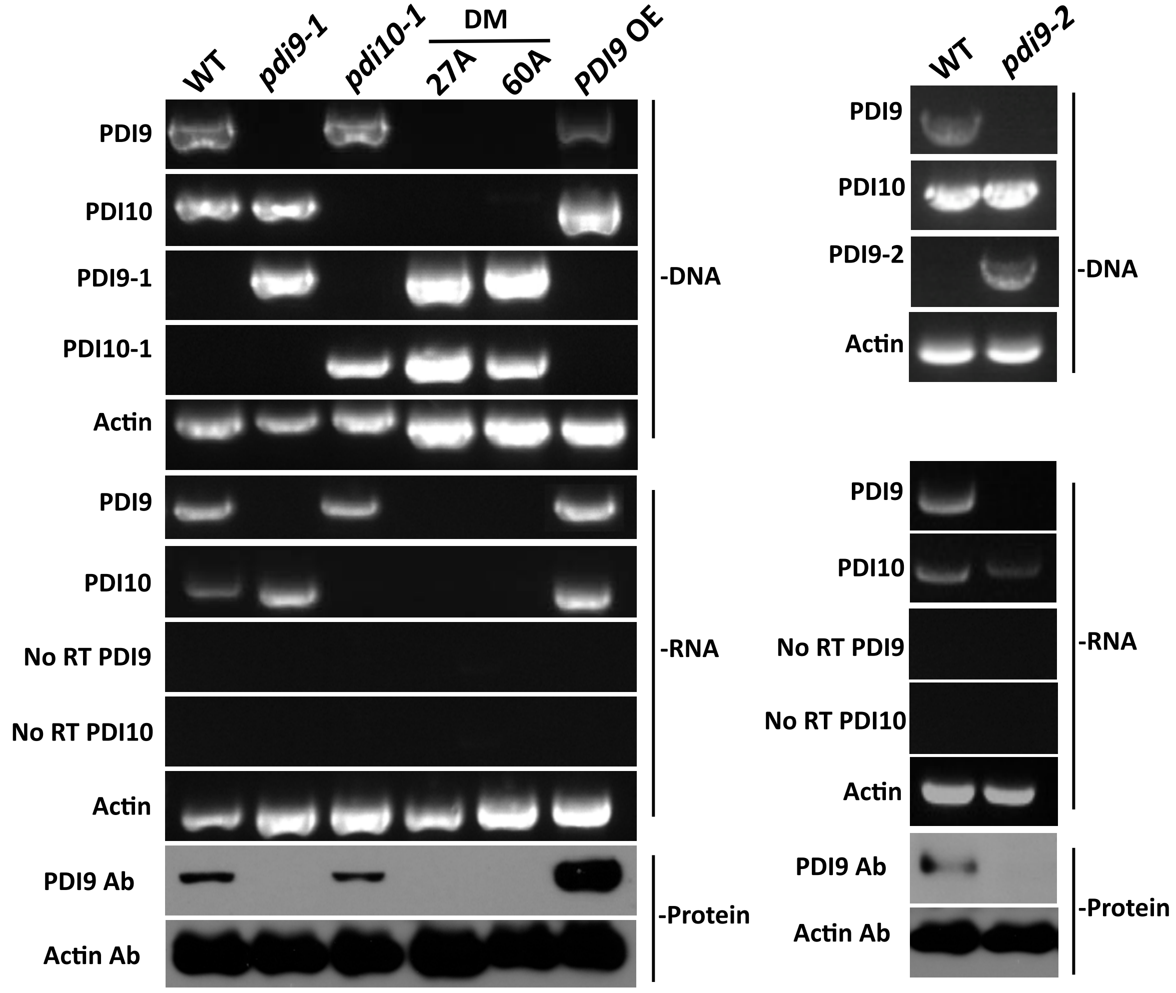

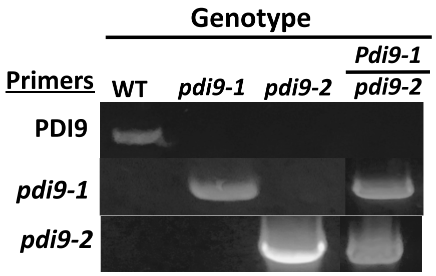

Supplementary Figure 2: Characterization and verification of the mutant genotypes and deficiency of gene expression relative to wild type (WT) by using genomic PCR (labeled 'DNA"), RT-PCR (labeled "RNA") and PDI9 immunoblot analysis (labeled "Protein"). The actin gene, mRNA and protein were used as internal standards. WT *PDI9*- and *PDI10*-specific primers were designed to flank the T-DNA such that no respective PCR product would be produced in the gene mutants. The T-DNA insertional mutants, *pdi9-1, pdi9-2,* and *pdi10-1* were determined to have the appropriate T-DNA insertion in their genomic DNA by PCR using gene and T-DNA specific primers. The *pdi9-1 pdi10-1* double mutant lines, 27A and 60A, have T-DNA insertions in both *PDI9* and *PDI10* genes. The *35S:PDI9* overexpressor (OE) in the WT background was included as a positive control. RT-PCR was used to confirm the deficiency of RNA in the mutants. A no reverse transcriptase was added as a negative PCR control. Immunoblotting with anti-PDI9 antiserum (PDI9 Ab) indicated the presence and absence of PDI9 protein in WT and the *pdi9* mutants, respectively: WT and the *pdi10* mutant have approximately equal amounts of PDI9 protein, whereas OE shows a relative abundance of PDI9. The *pdi9-1/pdi9-2* transheterozygote mutant was confirmed by genomic PCR (right panel) and sequencing the PCR product.
