# Supplementary material for: The Arabidopsis Protein Disulfide Isomerase Subfamily M Isoform, PDI9, Localizes to the Endoplasmic Reticulum and Influences Pollen Viability and Proper Formation of the Pollen Exine During Heat Stress
Source: Front Plant Sci. 2020 Dec 29;11:610052. doi: 10.3389/fpls.2020.610052 (PMC7802077; doi:10.3389/fpls.2020.610052)
Supplement: Supplementary file 3 [file Data_Sheet_3.pdf]

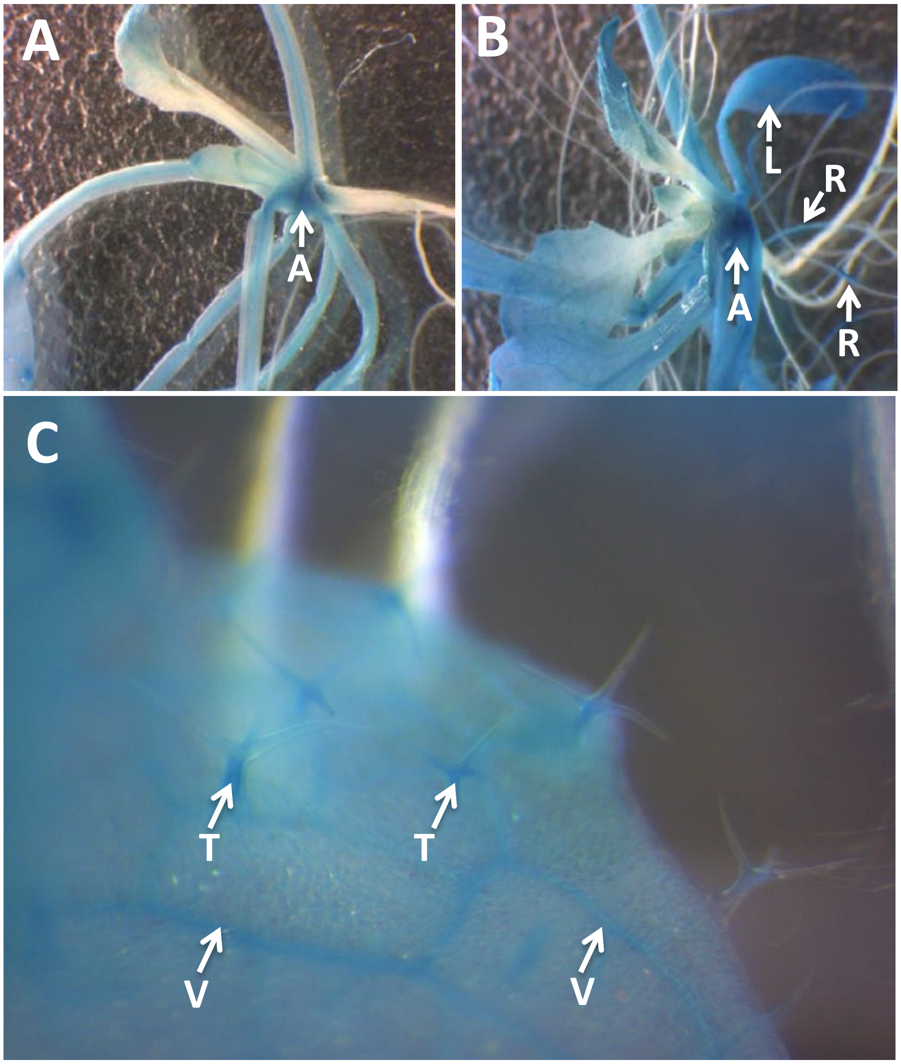

Supplementary Figure 3: Histochemical analysis of the expression of PDI-M gene promoters in 14-day-old Arabidopsis plants. *$PDI9_{promoter}$:GUS* **(A, C)** and *$PDI10_{promoter}$:GUS* ***(B)*** expression are shown. White arrows indicate areas of GUS staining and expression and are labeled T (trichomes), V (vascular system), R (roots), A (apical meristem) and L (leaf).
