# Supplementary material for: The Arabidopsis Protein Disulfide Isomerase Subfamily M Isoform, PDI9, Localizes to the Endoplasmic Reticulum and Influences Pollen Viability and Proper Formation of the Pollen Exine During Heat Stress
Source: Front Plant Sci. 2020 Dec 29;11:610052. doi: 10.3389/fpls.2020.610052 (PMC7802077; doi:10.3389/fpls.2020.610052)
Supplement: Supplementary file 4 [file Data_Sheet_4.pdf]

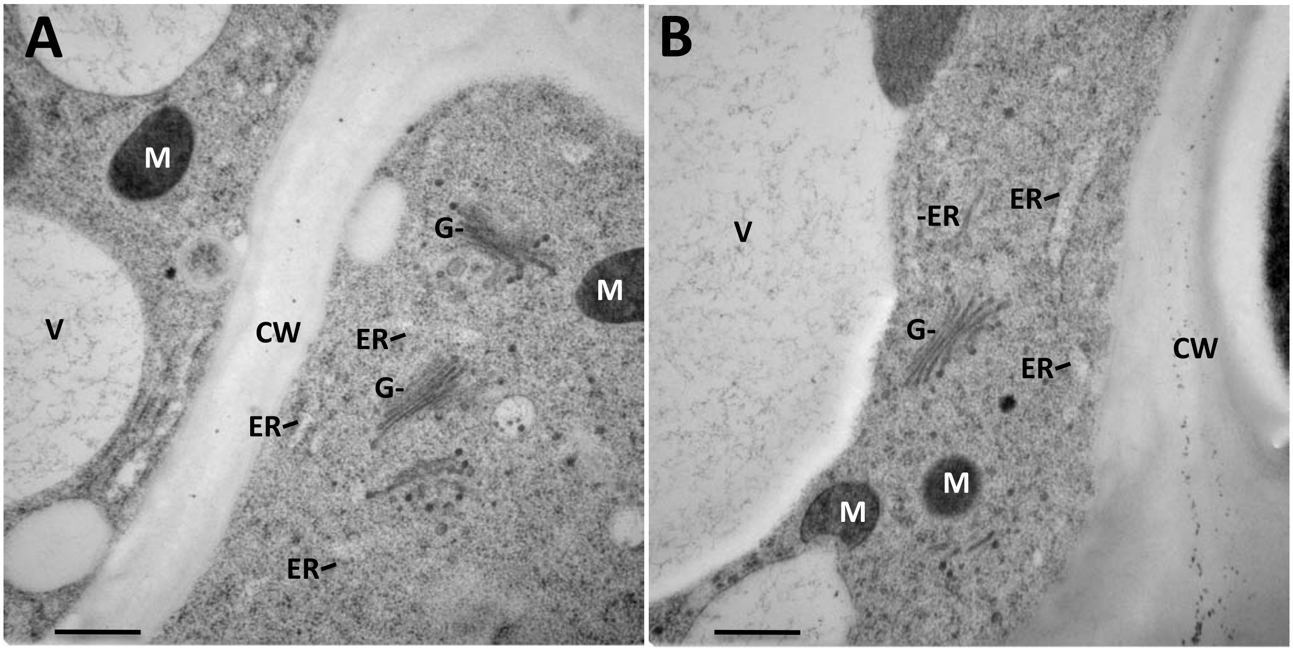

Supplementary Figure 4: TEM immunolabeling with the PDI9 antiserum (and 10 nm gold anti-rabbit secondary antiserum) on roots from the *pdi9-1* mutant (A) and the *pdi9-pdi10* mutant (B) indicates no non-specific labeling. Labels of subcellular structures are indicated for reference, ER, endoplasmic reticulum; cell wall, CW; mitochondrion, M; V, vacuole; G, Golgi apparatus. Scale bars are 500 nm.
